# Psychosocial Interventions and Wellbeing in Individuals with Diabetes Mellitus: A Systematic Review and Meta-Analysis

**DOI:** 10.3389/fpsyg.2017.02063

**Published:** 2017-12-05

**Authors:** Michaela C. Pascoe, David R. Thompson, David J. Castle, Zoe M. Jenkins, Chantal F. Ski

**Affiliations:** ^1^Institute of Sport, Exercise and Active Living (ISEAL), Victoria University, Melbourne, VIC, Australia; ^2^Peter MacCallum Cancer Centre, Melbourne, VIC, Australia; ^3^Department of Psychiatry, University of Melbourne, Melbourne, VIC, Australia; ^4^Department of Epidemiology and Preventive Medicine, Monash University, Melbourne, VIC, Australia; ^5^Mental Health Service, St. Vincent's Hospital, Melbourne, VIC, Australia

**Keywords:** psychosocial interventions, wellbeing, diabetes mellitus, systematic review, meta-analysis

## Abstract

**Purpose:** A number of studies, including systematic reviews, show beneficial effects of psychosocial interventions for people with diabetes mellitus; however, they have not been assessed using meta-analysis. The purpose of this meta-analysis of randomized controlled trials is to investigate the effects of psychosocial interventions on depressive and anxiety symptoms, quality of life and self-efficacy in individuals with diabetes mellitus.

**Methods:** The databases Pubmed, MEDLINE, CINAHL, PsycINFO, Scopus, Web of Science and SocINDEX were searched with no year restriction. Eligible studies were randomized controlled trials published in English that included individuals diagnosed with diabetes mellitus, aged 18 years or above, who engaged in a psychosocial intervention, with outcome measures addressing depressive or anxiety symptomology, quality of life or self-efficacy. Eligible studies needed to compare the intervention to usual care. Study selection was completed using Covidence and meta-analysis was undertaken using Comprehensive Meta-Analysis software.

**Results:** Seven studies were included in the meta-analysis. Five studies investigated the effects of psychosocial interventions and showed a medium to large benefit for depressive symptoms (*SMD*: −0.70; CI: −1.27, −0.13) which persisted at follow up (*SMD*: −1.54, *CI*: −2.97, −0.12). Similar results were not seen immediately post-intervention in the three studies that assessed anxiety symptoms (*SMD*: −0.30; CI: −0.69, 0.10); however, a medium beneficial effect was seen at follow up (*SMD* = −0.61, *CI* = −0.92 to −0.31). Small benefits were seen in the three studies assessing quality of life outcomes (*SMD*: 0.30, *CI*: 0.06, 0.55). No benefit was seen in the two studies assessing self-efficacy (*SMD*: 0.23, *CI*: −0.11, 0.57).

**Conclusions:** The results of the current study provide preliminary evidence that psychosocial interventions, compared to usual care, reduce depressive symptoms, and may improve quality of life in individuals with diabetes. However, only a few studies were included and the clinical significance of these findings is unknown.

## Introduction

The worldwide burden of diagnosed diabetes mellitus (DM) was approximately 422 million in 2014 (NCD-RisC, 2016). This number is expected to reach 592 million by 2035 (Guariguata et al., 2014) and an additional 174 million individuals are estimated to have undiagnosed DM (Beagley et al., 2014). In 2012, the total costs associated with the treatment of DM in the United Kingdom was £13.7 billion (Kanavos et al., 2012) and $245 billion in the United States (American Diabetes Association, 2013). Given the high prevalence of DM and associated impact on individuals and communities, it is important to understand the factors influencing wellbeing to achieve the best possible quality of life (QoL) for individuals with DM.

One factor affecting QoL in individuals with DM is the development of depression and anxiety. Depressive and anxiety disorders are highly prevalent among individuals with chronic disease (Moussavi et al., 2007), including DM. Clinical depression affects approximately 12% of individuals with Type I diabetes and 19% of individuals with Type II diabetes (Roy and Lloyd, 2012), while generalized anxiety disorder affects approximately 14% of individuals with DM (Grigsby et al., 2002). Furthermore, up to 31% of people with DM experience sub-clinical levels of depression (Anderson et al., 2001) and up to 40% experience sub-clinical levels of anxiety (Grigsby et al., 2002), which also negatively influence health outcomes and management behavior (Gonzalez et al., 2008). Depression and anxiety are highly comorbid with one another, sharing a similar etiology and neurobiology (Neale and Kendler, 1995). Anxiety is the single strongest predictor of depression onset (Mathew et al., 2011), and while neurochemical differences exist between the conditions, recent studies suggest that depression and anxiety may be overlapping syndromes, existing on a continuum (Baldwin et al., 2002). Aside from impairing overall QoL and wellbeing, DM-associated depression impairs functional ability (Smith and Schmitz, 2014) and compromises glycaemic control (Anderson et al., 2002), whilst being associated with increased risks of hospitalization (Davydow et al., 2013), dementia (Katon et al., 2012) and mortality (Hofmann et al., 2013). Accordingly, the incorporation of psychological wellbeing in the management of DM is commonplace in national standards of care (Craig et al., 2011).

Social support is an important predictor of outcomes in DM (Strom and Egede, 2012). Social support from peers and other individuals is associated with improved metabolic control (Trento et al., 2001), clinical outcomes (Strom and Egede, 2012), increased physical activity (Keyserling et al., 2002), DM knowledge (Gilden et al., 1992), adherence to healthy behavior regimes (Strom and Egede, 2012) and decreased DM related distress (Baek et al., 2014). Reduced social support and depression often coexist, with the two sharing a bidirectional relationship (Lett et al., 2005). A low level of social support is an important contributing factor to DM-related depression, whilst depression reciprocally contributes to lowered levels of social support (Sacco and Yanover, 2006). The interplay between social support and depression indicates the importance of utilizing interventions that address social support when treating depression in individuals with DM. Psychological interventions such as cognitive behavioral therapy have been shown to be effective in the treatment of depression in DM (Baumeister et al., 2012). A systematic review of eight studies using various psychological interventions, including cognitive behavioral therapy and psychodynamic supportive therapy, demonstrated that these reduced depression severity and remission rates in both the short and medium term, in individuals with DM (Baumeister et al., 2012). Therefore, both psychological interventions and social support are important in the treatment of depression.

In non-DM populations, psychosocial interventions have been shown to decrease depressive and anxiety symptoms (Jacobsen and Jim, 2008; Forsman et al., 2011a,b). Indeed, our group has previously evaluated the effect of psychosocial interventions on depression and anxiety symptoms in individuals with cardiovascular disease. In a meta-analysis of six eligible randomized controlled trials (RCTs), we found a small significant benefit for psychosocial interventions on depressive symptoms (Ski et al., 2015).

Harkness et al. (2010) explored the impact of a diverse range of lifestyle intervention to manage diabetes or psychological intervention to manage mental health in people with DM, using systematic review and meta-analysis. The authors reported that no specific characteristics of lifestyle or psychological interventions predicted substantial benefits in physical and mental health outcomes. Harkness et al. (2010), however did not restrict their analysis to psychosocial interventions, defined as any intervention that combines both psychological and social components (Thompson and Ski, 2013). Few studies have been conducted to evaluate interventions comprising both psychological and social support enhancing components for depression and anxiety in people with DM. One systematic review included 10 qualitative studies, including psychosocial interventions, aimed at reducing depression in individuals with DM (Kok et al., 2015). While the results of this review study showed promising effects (Kok et al., 2015), no meta-analysis has been undertaken of RCTs including strictly psychosocial interventions. Therefore, the aim of the present study was to assess the effects of psychosocial interventions in the context of DM. Specifically, we aimed to conduct a systematic review and meta-analysis of RCTs investigating the effects of psychosocial interventions on depression and anxiety as well as QoL and self-efficacy, compared to usual care (UC) in individuals with DM.

## Materials and methods

### Data sources and search strategy

This study was conducted following the Preferred Reporting Items for Systematic Reviews and Meta-Analyses (PRISMA) guidelines/protocol (Moher et al., 2010). A prospective protocol for the systematic review was not previously published. For two articles authors were contacted to request clarification as to whether group assignment was randomized (Trozzolino et al., 2003) or if the intervention delivered incorporated a social component or not (Simson et al., 2008). These authors did not respond and we were unable to determine if the studies met our inclusion criteria. Thus, we could not include these studies in the review or meta-analysis.

Eligible studies were randomized controlled trials (RCTs) published in English that included individuals diagnosed with DM only (no requirement for a diagnosis of depression or anxiety) who engaged in a psychosocial intervention compared to usual care. Eligible studies were required to compare the intervention to usual care on at least one of the following outcomes: depressive or anxiety symptomology, QoL or self-efficacy. Other outcomes collected were body mass index (BMI), hemoglobin A1c (HbA1c), social support, and fasting blood glucose (FBG). A psychosocial intervention is defined as any intervention that combines psychological and social components (Thompson and Ski, 2013). Psychological components would be those pertaining to an individual's behavior and mind inclusive of cognition and emotion, e.g., cognitive behavioral therapy, motivational interviewing, or psycho-education. Social components would be those pertaining to social support or building interpersonal skills. However, it is acknowledged that these components may vary in the literature. Review papers, non-randomized trials, case series, and dissertations were excluded. Eligible studies included participants over 18 years of age. Interventions could be administered by any personnel and be implemented through a range of modes, e.g., face to face, telephone, telehealth or online. The primary outcomes were depressive or anxiety symptoms. Secondary outcomes were QoL and self-efficacy.

Searches were undertaken in January 2016 and updated in March 2017. Articles were obtained by searching electronic databases: PubMed, MEDLINE, CINAHL, PsycINFO, Scopus, Web of Science and SocINDEX. Conference abstracts or trial databases were not searched as we aimed only to include complete RCTs that provided sufficient data for inclusion and risk of bias assessment. Databases were searched for articles with no year restriction and containing the specific title or MeSH words, “Diabet^*^,” or “glucose,” or “hyperglycemia,” or “hypoglycemia,” or “glycohemoglobin,” or “metabolic syndrome,” or “insulin” and the title/abstract or MeSH words “psych^*^,” or “motivational interviewing,” or “motivational behavior,” or “motivational behavior,” or “behavior interviewing,” or “behavior interviewing,” or “behavior change,” or “behavior change,” or “motivational change,” or “non-invasive change,” or “intervention,” and the title/abstract or MeSH word “depress^*^,” or “anxi^*^,” or “melancholia,” or “dysthymia,” or “mood,” or “quality of life,” or “self-efficacy,” or “coping,” or “stress.” In an attempt to identify as many potentially eligible studies as possible, the term “RCT” was not a filter in the initial search strategy. We deliberately used broad search terms in an attempt to capture as many interventions that might contain both a psychological and social component as possible. While this method of searching also identified many irrelevant studies, it ensured that we captured as many potentially eligible studies as possible. The terms “psych^*^” and “intervention,” for example were broad enough to capture both relevant and irrelevant studies. We felt that the search term “non-invasive change” for example was likely to capture studies with an intervention containing a psychological or social support aspect, as this term has been used in previous literature to describe “any treatment or action, based on clinical judgment and knowledge, that healthcare professionals (physicians, nurses, psychologists, physiotherapists, occupational therapists, dieticians) perform to enhance patient well-being or quality of life (Rueda et al., 2011).” The terms “behavior change,” and “motivational change” have been used in the literature to describe interventions containing a psychological aspect (Wade et al., 2009; Michie et al., 2011). Conference abstracts and technical reports were also excluded as they were not likely to include the detailed information required for assessment of bias or meta-analysis inclusion.

### Study selection

Sourced studies were imported into Covidence online software (https://www.covidence.org) and assessed for full text eligibility based on title/abstract by two independent reviewers (MCP, ZMJ); disagreements were resolved through discussion or by consulting a third reviewer (CFS).

### Data extraction and quality assessment

Relevant data were extracted from each study using a predesigned data extraction form, including study design, country undertaken, aims, ethical information, studied outcomes, sample size and participant characteristics. Intervention characteristics included delivery method, components, personnel involved, duration and follow up. The mean (M), standard deviation (SD), and sample size (n) were extracted. Study authors were contacted if published data were incomplete or unclear. In two studies the authors were contacted and they advised that individuals collecting outcome measures were blinded (D'Eramo Melkus et al., 2010; Rosland et al., 2015). In two studies relevant means and standard deviations for outcomes of interest were not reported in the text and were provided by the authors upon request (completers) (D'Eramo Melkus et al., 2010; Stoop et al., 2015). Data were extracted independently by two reviewers and disagreements were resolved through discussion or by consulting a third reviewer.

Methodological quality of the studies was assessed independently by two reviewers using the Cochrane Collaboration's risk of bias assessment tool (Cochrane Collaboration, 2011). Due to the nature of the studies reviewed, the blinding of participants and personnel (administering the intervention) domain was not assessed in this review. To best capture the current state and quality of research in this field, papers were not included or excluded based on quality assessment, and thus all eligible articles were included. Grades of Recommendation, Assessment, Development and Evaluation (GRADE) were assessed using the GRADE working group recommendations as published in the Cochrane Handbook (Cochrane Collaboration, 2011). We considered five factors when assessing the quality of evidence: (1) risk of bias, (2) heterogeneity, (3) population, intervention, comparison, outcomes (PICO) (4) precision, and (5) publication bias (Cochrane Collaboration, 2011).

### Summary measures

For the meta-analysis we report the raw difference in means when the outcome is reported on the same meaningful scale in all studies The standardized mean difference (*SMD*) was used in place of mean difference when studies included in the meta-analysis used different outcome measures and thus the different scales used were not comparable in raw form (Borenstein et al., 2009). The *SMD*, is where the mean difference in each study is divided by the standard deviation (SD) to create an index that is comparable across studies (Borenstein et al., 2009). The sample estimate of the *SMD* was Hedges G (*g*), which corrects for bias due to small sample size. A small effect is considered 0.2, medium 0.5 and large 0.8 (Nakagawa and Cuthill, 2007; Borenstein et al., 2009). In studies where multiple outcomes were used to measure the depression, anxiety, or QoL outcomes, composite scores using the mean of the various outcomes were used.

### Data synthesis and analysis

Meta-analysis was undertaken using Comprehensive Meta-Analysis (CMA) software version 3 (CMA, Biostat, USA). The primary analysis compared the effect of the intervention (psychosocial intervention) with UC groups on depression, anxiety, QoL and self-efficacy scores. Other extracted outcomes were body mass index (BMI), hemoglobin A1c (HbA1c), social support, and fasting blood glucose (FBG). The *Q* statistic was used to assess if effect size varied across studies and the *p*-value used to determine statistical significance was 0.10. The proportion of the observed variance reflects differences in true effect sizes rather than sampling error as shown by the *I*^2^ statistic (Borenstein et al., 2009). A funnel plot was used to ascertain any publication bias, as shown in Supplementary Figure 1. Sensitivity analyses were performed using “one study removed analysis” to detect whether the observed effect was unduly influenced by any single study. All studies were sampled from a universe of possible studies defined by the inclusion/exclusion. A random effects model was used in all analysis, weighting the studies based on the sample size/standard error. When pre-post correlations were not reported in the published paper, we conducted sensitivity analysis using a correlation of 0 and a correlation of 0.9, and found the results of our primary outcomes of interest to be the same, thus we used a 0 correlation for all analyses.

## Results

**Figure 1:**
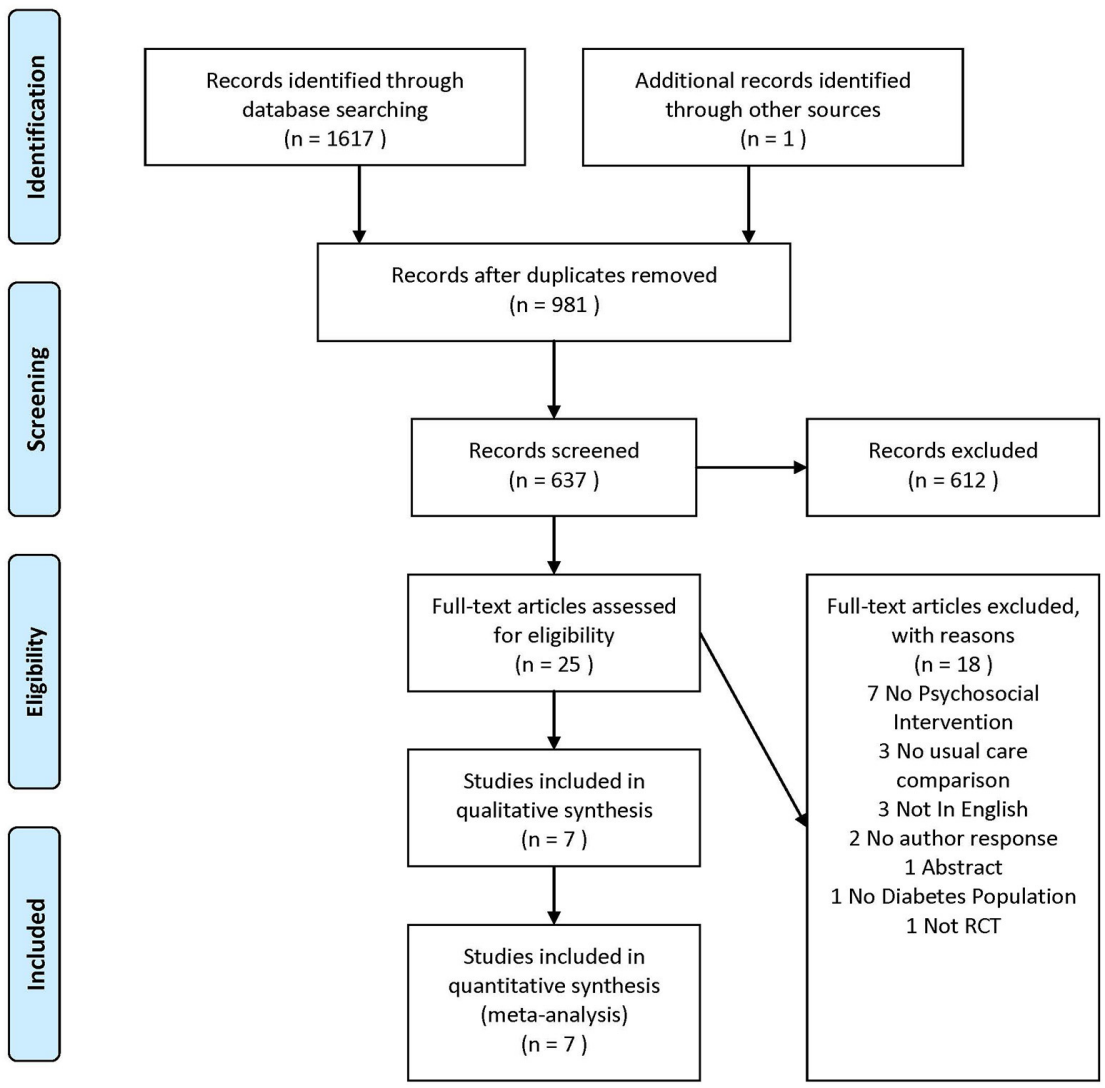
Flow Chart Showing the Retrieval Process of Trials included in the Meta-analysis.

We initially retrieved 1,618 papers, 981 were duplicates, leaving 637 for screening. 612 were excluded from title/abstract screening leaving 25 for full text review. Seven of these were included in the study. The PRISMA flow diagram illustrates the reasons for study exclusions (Figure 1).

### Study descriptions

Table 1 shows that the RCTs were two-group, parallel designs. Sample sizes ranged from 18 to 111 and participant ages ranged from 45 to 64 years. The percentage of women ranged from 23 to 100%. In all but one study (Kuijer et al., 2007) participants were diagnosed with type II diabetes. In one trial, 56% of participants were diagnosed with DM and 44% with asthma; only data relating to individuals with DM have been included here (Kuijer et al., 2007).

**Table 1:** Characteristics of the trials included in the meta-analysis.

**References**	**Country**	**Study Design**	**Participants**	**Intervention Type and N at baseline**	**Comparison Group and N at baseline**	**Time of Assessment**	**Outcome Measures and N at post intervention**	**Relevant differences Between Groups**	**Follow up (from intervention completion)**	**Journal**
D'Eramo Melkus et al., 2010	USA	Parallel group *P*-values both ITT and completers – M ± SD requested from author	African American women with type 2 diabetes (*n* = 109)	Cognitive behavioral diabetes self-management training and coping skills training (*n* = 57; M age 47 ± 9; Female = 57 [100%])	Usual Care (*n* = 52; M age 45 ± 10; Female = 52 [100%])	Pre-post Intervention, follow up (QoL not assessed post intervention)	CCEI; PAID; DCP; DSEQ; DKT; (MOS)-SF-36; MHCCQ; FBG; BP; HbA1c; LDL-C; HDL-C; TG (intervention *N* = 40; Control *N* = 37)	Intervention associated with lower diabetes-related emotional distress and higher perceived family support at 24 months follow up. No difference between groups in somatic anxiety In study attendees (*n* = 32 did not attend any sessions) lower levels of diabetes-related emotional distress and higher levels of QOL were seen at 24 m. No difference between groups in somatic anxiety	3, 6,9,21 months	Biol Res Nurs.
Kuijer et al., 2007	The Netherlands	Parallel group ITT (replaced with baseline data)	Adults diagnosed with diabetes (*n* = 55 [56%]) or asthma (*n* = 70 [44%])	Self-management intervention (diabetes) (*n* = 26; M age 45 ± 12; Female = 12 [46%])	Usual Care (diabetes) (*n* = 16; M age 38 ± 11; Female = 7 [44%])	Pre-post follow up (post intervention assessments at 2 weeks post intervention completion)	C-QoL; SF-12, SESM, PCS, LOT, SDSCA (intervention *N* = 23; Control *N* = 15)	The intervention had no significant effect on any of the measured outcomes	6 months (intervention *N* = 20; Control *N* = 12)	Psychol Health
Moncrieft et al., 2016	USA	Parallel group ITT (method not stated)	Overweight/obese adults with Type 2 diabetes (*n* = 111)	CALM-D intervention (*n* = 57; M age 54 ± 8; Female = 37 [64%])	Usual Care (*n* = 54; M age 54 ± 6; Female = 42 [77%])	Pre, mid, and post intervention	*Weight HbA1c, BDI* (intervention *N* = 41; Control *N* = 46)	The intervention decreased weight and depressive symptoms.	None	Psychosom Med
Penckofer et al., 2012	USA	Parallel group- *P*-values both ITT and completers – M ± SD completers only	Women diagnosed with depression and type 2 diabetes (*n* = 74)	Psychoeducational (SWEEP) group therapy (*n* = 38; M age 55 ± 9; Female = 38 [100%])	Usual Care (*n* = 36; M age 54 ± 8; Female = 36 [100%])	Pre Intervention, follow up (post intervention assessments at approximately 4 weeks post intervention completion). Two short booster sessions between post intervention and follow up	*CES*-*D, STAI*, STAGI*, DIS*, QoLd-III, SF-12, HbA1c (intervention *N* = 29; Control *N* = 36)	The intervention decreased depression, trait anxiety, anger expression, improved QoL, psychological and spiritual satisfaction	3 months (intervention *N* = 26; Control *N* = 34)	Ann Behav Med
Rosland et al., 2015	USA	Parallel group - M ± SD completers only	African-American or Latino adults with type 2 diabetes (*n* = 183)	Empowerment-based community health worker-led intervention (n = 84; M age 52 ± 10; Female = 42 [75%])	Usual Care (*n* = 99; *M* age 56 ± 12; Female = 35 [67%])	Pre-post Intervention	PHQ-9, DCP, HbA1c, (intervention *N* = 56; Control *N* = 52)	The intervention improved HbA1c. Social support at baseline associated with greater change in HbA1c independent of group assignment Overall depressive symptoms did not significantly change while participants received the 6-month intervention.	None	Patient Educ Couns
Stoop et al., 2015	The Netherlands	Parallel group	Adults diagnosed with type 2 diabetes (52%) (*n* = 24) or asthma (48%) (*n* = 22)	Disease Management program (*n* = 12; M age; Female = [%])	Usual Care (*n* = 12; *M* age ± SD; Female = [%])	Pre-post Intervention, follow up	GAD-7, PHQ-9, MINI (intervention *N* = 20; Control *N* = 18)	The intervention associated with decreased depressive and anxious symptoms post intervention and decreased anxious symptoms at follow up	6 months (intervention *N* = 18; Control *N* = 18)	J Affect Disord
Yu Huang et al., 2015	Taiwan	Parallel group- M ± SD completers only	Adults with type 2 diabetes in Taiwan (*n* = 65)	CBT plus MET (*n* = 31; M age 55 ± 10; Female = 15 [48%])	Usual Care (*n* = 30; M age 58 ± 10; Female = 17 [57%])	Pre-post Intervention, follow up	CES-D, SF-36, HbA1C (intervention *n* = 31; Control *n* = 30)	The intervention was associated with decreased depression and increase QoL at post intervention and follow up, as well as decreased BMI, fasting glucose and improved HbA1C.	3 months (intervention *n* = 31; Control *n* = 30)	Qual Life Res

*PHQ-9, 9-item Patient Health Questionnaire; PAID, 25-item Problem Areas in Diabetes Survey; BP, Blood Pressure; C-Qol, Cantril's ladder Quality of life Scale; CALM-D, Community Approach to Lifestyle Modification for Diabetes; Crown-Crisp eGFR, estimated glomerular filtration rate; CCEI, Experimental Index; DCP, Diabetes Care Profile; DSEQ, Diabetes Self-Efficacy Outcomes Expectancies Questionnaire; DKT, Diabetes Knowledge Test; DIS, Diagnostic Interview Schedule; FBG, Fasting blood glucose; GAD-7, General Anxiety Disorder questionnaire; HbA1c, Glycated haemoglobin; HDL-C, High-density lipoprotein cholesterol; LOT, Life Orientation Test; LDL-C, Low-density lipoprotein cholesterol; SF-36 = (MOS)-SF-36, Medical Outcomes Study; MHCCQ, Modified Health Care Climate Questionnaires; MINI, Modules of the Mini-International Neuropsychiatric Interview; PCS, Proactive Coping Subscale of the Proactive Coping Inventory; SESM, Self-efficacy beliefs regarding self-management scale; SF-36, Short form health survey; SF-12, Short form health survey; STAI, State–Trait Anxiety Inventory; STAGI, State–Trait Anger Expression Inventory; SDSCA, Summary of Diabetes Self-Care Activities questionnaire; CES-D, The Centre for Epidemiological Studies-Depression; TG, Triglyceride; QoLd-II, Quality of Life Index—Diabetes III Version*.

Depressive and anxiety symptoms were assessed in all but one study, which included a QoL outcome (Kuijer et al., 2007). Depressive symptoms were assessed using the Centre for Epidemiologic Studies Depression scale (CES-D) in two studies (Penckofer et al., 2012; Huang et al., 2015), the 9-item Patient Health Questionnaire (PHQ-9) in two studies (Rosland et al., 2015; Stoop et al., 2015), and the Beck Depression Inventory II (BDI) in one study (Moncrieft et al., 2016). Anxiety was measured in three studies and was assessed using the Crown-Crisp Experiential Index (CCEI) somatic anxiety subscale in one study (D'Eramo Melkus et al., 2010), the State Trait Anxiety Inventory (STAI) in one study (Penckofer et al., 2012) and the 7-item Generalized Anxiety Disorder questionnaire (GAD-7) in one study (Stoop et al., 2015).

QoL was measured in three studies and was assessed using the Short-Form 12-item health survey (SF-12) in two studies (Kuijer et al., 2007; Penckofer et al., 2012) and the Short-Form 36-item health survey (SF-36) in one study (Huang et al., 2015). Additionally, the Medical Outcomes Study (MOS) survey was used in one study (D'Eramo Melkus et al., 2010) the QoL Index Diabetes III Version (QoLd-III) in another study (Penckofer et al., 2012) and Cantril's ladder QoL scale (C-QoL) in another (Kuijer et al., 2007).

Self-efficacy was measured in two studies. One study measured individual's confidence in their ability to perform a series of regimen behaviors using the Diabetes Self-efficacy Outcomes Expectancy Questionnaire (DSEQ) (D'Eramo Melkus et al., 2010) while one study measured self-efficacy using a Self-efficacy beliefs scale regarding self-management (SESM) and the Summary of Diabetes Self-Care Activities questionnaire (SDSCA) (Kuijer et al., 2007).

Other outcomes included social support (D'Eramo Melkus et al., 2010; Rosland et al., 2015) measured using a subscale of the Diabetes Care Profile (DCP) (D'Eramo Melkus et al., 2010; Rosland et al., 2015), HbA1c (D'Eramo Melkus et al., 2010; Penckofer et al., 2012; Huang et al., 2015; Rosland et al., 2015; Moncrieft et al., 2016), FBG (D'Eramo Melkus et al., 2010; Penckofer et al., 2012; Huang et al., 2015) and BMI (D'Eramo Melkus et al., 2010; Huang et al., 2015).

Two studies had an intervention duration of 12 weeks or 3 months (D'Eramo Melkus et al., 2010; Huang et al., 2015), two had an intervention duration of 8 weeks (Kuijer et al., 2007; Penckofer et al., 2012), one of 6 months (Rosland et al., 2015) and another two studies of 12 months (Stoop et al., 2015; Moncrieft et al., 2016). The psychosocial interventions in each study varied in their components, frequency and duration as shown in Table 2.

**Table 2:** Characteristics of the psychosocial interventions.

**References**	**Setting**	**Personnel delivering treatment**	**Psychological component**	**Social support component**	**Topics addressed/components**	**Delivery Setting**	**Mode of delivery**	**Invention Duration**
D'Eramo Melkus et al., 2010	Community health center	Clinical psychologist or psychiatric nurse	Cognitive behavioral training/Intervention	Communication training	Diabetes education; behavior change; Multiple life roles and the stress cycle; problem identification and explorations; problem-solving strategies; managing stress; communication - active listening, assertiveness, and refusal techniques.	Group	In person	12 weeks–2 h/week/6weeks then 1/week/5weeks
Huang et al., 2015	Hospital outpatients	Psychotherapist	CBT	Building /maintain social support networks and Linking to social services	Dietary education, complication prevention, lifestyle behavior change, and coping stress management, appraisal skills and problem-solving techniques training, enhancing motivation for better self-achievement and attending activities of the individual's social network, improving their assertiveness and access to social support	Group	In person	3 months 80min/week/12weeks (4 sessions MET, 8 session CBT)
Kuijer et al., 2007	Hospital outpatients	Nurse	Behaviour modification	Building/maintaining social support networks	Maintaining a good physical condition, preventing exacerbation, recognition of first symptoms and taking adequate action, coping with negative emotions in relation to being chronically ill, giving and seeking social support from partner, neighbors and colleagues	Group	In person	8 weeks−2 h/biweekly/8 weeks Plus 2 h 4weeks after previous session
Moncrieft et al., 2016	Clinical	Trained therapists	Psycho-education, stress management, goal setting	Increasing social support, working with social cues	Topic covered included: CALM-D goals, deep breathing, using fat counter, identify ways to eat less fat, food logging, life-style activity, preventing injury, physical activity goal, types of negative thinking, emotional eating, doctor patient communication, stress effects on the body, pleasurable activities, calorie goals, food pyramid, rate your plate, calories and weight loss, body language, listening techniques, identifying and changing food and activity cues, social support, 5 steps of problem solving, action plans, practice four keys to eating out, identify potential slips, identify negative thoughts, challenge negative thoughts, FITT principles, heart rate monitoring, target heart rates, 3As of stress management, unavoidable stressors, assertiveness, social cues, set personal goals, develop action plan to maintain motivation	Group and individual	In person	12 months 1.5–2 h/week/4 weeks then biweekly/4 weeks then 1 × month/9 months
Penckofer et al., 2012	Academic center	Nurse supervised by a clinical psychologist	CBT	Conflict resolution and relationship building	Education about recognizing the signs/symptoms of depression and other moods, the relationship between moods, metabolic control and self-care behaviors, and the management of depression, anxiety, and anger using CBT, progressive muscle relaxation, reduce anger, build relationships, manage anxiety, resolve conflict, learn assertive skills, and build positive attitudes	Group	In person	8 Weeks plus 2 booster within 3 months−1 h/week/8weeks plus 2 booster sessions within 6 months
Rosland et al., 2015	Community health center	Community health worker	Behaviour modification	Communication training and linking to social services	Diabetes education classes, participants' specific self-management goals family health advocates helped participants improve their patient-provider communication skills and facilitated necessary referrals to other service systems	Group and individual	In person	6 months – 2 h/fortnight/22 weeks Plus 2 60 m home visits plus 1 clinic visit
Stoop et al., 2015	Primary care centers	Psychologist	Psycho-education and CBT	Social skills training	Education about symptoms, causes, prevalence and course of depression/anxiety, information about the link of lifestyle with symptoms of depression/anxiety. Training in behavioral activation, social skills training, and relapse prevention. Advice to meet the general practitioner to discuss antidepressant or antianxiety medication options and booster sessions.	Individual	In person	12 months - 1.5–2 h/1 × wk 4 wks, 1 × bi wk 4 weeks, 30 m/week/4 weeks then 30 m/week/10 weeks then 6 sessions/6 months. (4 sessions psycho-education, 10 session Coping, 6 sessions medication/booster)

*CBT, Cognitive Behavioral Therapy; CALM-D, Community Approach to Lifestyle Modification for Diabetes; MET, Motivation Enhancement Therapy*.

### Risk of bias

Table 3 shows that on each of the domains the vast majority of the included RCTs were rated as having a low or unclear risk of bias, which is insufficient to justify downgrading the level of evidence. However, as seen below in the meta-analysis results and in Supplementary Table 1, heterogeneity exists between study outcomes for depression symptoms at post intervention and QoL at 3 months follow up. This heterogeneity appears to result from differences in measurement tools and populations studied, making reliable sub-group analysis difficult. In terms of PICOs, we consider the population, interventions, comparison and outcomes to be sufficiently direct to address the question at hand. In terms of precision, we consider the sample size to be sufficiently large for the depression (*n* = 340) and QoL (*n* = 267) outcomes. For anxiety symptoms, the total sample was only *n* = 151. For self-efficacy, the total sample size was only *n* = 140. Finally, in terms of publication bias, a funnel plot of depressive symptoms indicated potential publication bias. There were too few studies of anxiety symptoms, QoL and self-efficacy to assess funnel plots for this outcome reliably. Given the above considerations, we suggest that the GRADE of evidence should be downgraded to moderate from high for all outcomes. Table 4 shows the tools used to assess depression, anxiety, self-efficacy and quality of life in the included studies. In meta-analyses where the listed tool reads as ‘combined,’ the tools listed in Table 4 were combined and assessed together in the analysis, for that outcome.

**Table 3:** Risk of bias assessment for included studies.

**References**	**Random sequence generation**	**Allocation concealment**	**Blinding of outcome assessment**	**Attrition bias**	**Selective reporting**	**Other bias**
D'Eramo Melkus et al., 2010	Low	UC	Low	UC	UC	High
Kuijer et al., 2007	UC	UC	UC	Low	UC	Low
Penckofer et al., 2012	Low	UC	High	UC	Low	Low
Rosland et al., 2015	UC	UC	Low	High	UC	High
Stoop et al., 2015	Low	Low	High	UC	High	High
Huang et al., 2015	Low	UC	UC	UC	UC	Low
Moncrieft et al., 2016	Low	Low	Low	Low	High	Low

*UC, Unclear; Random sequence generation and allocation concealment Two studies did not state the method of randomization (Kuijer et al., 2007; Rosland et al., 2015). Random sequencing occurred, but with no mention of if allocation concealment was used, in five studies (Kuijer et al., 2007; Penckofer et al., 2012; Huang et al., 2015; Rosland et al., 2015). In one study, participants were randomized after baseline testing (D'Eramo Melkus et al., 2010). Blinding of outcome assessment In two studies blinding of outcome assessment did not occur (Penckofer et al., 2012; Stoop et al., 2015). In two studies the authors were contacted and advised that individuals collecting outcome measures were blinded (D'Eramo Melkus et al., 2010; Rosland et al., 2015). One study failed to provide sufficient information to determine this (Kuijer et al., 2007). Attrition bias Attrition occurred in all studies. One study performed and reported data for intention-to-treat analysis only, using baseline data to replace missing data (Kuijer et al., 2007). In two studies relevant means and standard deviations for outcomes of interest were not reported in the text and were provided by the author upon request (completers) (D'Eramo Melkus et al., 2010; Stoop et al., 2015). In three studies, in text means and standard deviations were provided only for study completers (Penckofer et al., 2012; Huang et al., 2015; Rosland et al., 2015). Means and standard deviations for study completers only was used in the meta-analysis, except for one study (Kuijer et al., 2007) where only intention-to-treat data was presented. In one study, risk-of-bias due to attrition was listed as high, as five participants originally randomized to the control group were switched to the intervention group and data was reported as treated rather than as randomized. Additionally, outcomes were reported for 108 completers from the 183 randomized and the reasons for drop out are not listed (Rosland et al., 2015) Selective reporting Four studies had previously published protocols (Stoop et al., 2011, 2015; Penckofer et al., 2012). In two of these outcomes listed in the protocol paper were not reported in the trial paper (Stoop et al., 2015; Moncrieft et al., 2016). Protocols were not available for the remaining four studies (Kuijer et al., 2007; D'Eramo Melkus et al., 2010; Huang et al., 2015; Rosland et al., 2015). Other bias In one study baseline somatic anxiety scores were higher in participants in the experimental group compared to the UC group (D'Eramo Melkus et al., 2010). In one study, five of the participants originally randomized to the UC group were switched to the intervention group (Rosland et al., 2015)*.

**Table 4:** List of studies and tools used in meta-analysis to examine depression, anxiety, or quality of life.

**Study**	**Depression**	**Anxiety**	**Self-Efficacy**	**QoL**
**SCALES USED**				
D'Eramo Melkus et al., 2010		CCI	DSEQ	
Huang et al., 2015	CES-D			SF-36 (Physical, Mental Function)
Kuijer et al., 2007			SDSCA, SESM	C-QoL, SF-12 (Physical, Mental Function)
Penckofer et al., 2012	CES-D	STA-S, STA-T		QoL-III (Family, health, overall, psychological socioeconomic), SF-12 (Physical, Mental Function)
Stoop et al., 2015	PHQ-9	GAD-7		
Rosland et al., 2015	PHQ-9			
Moncrieft et al., 2016	BDI			

*PHQ-9, 9-item Patient Health Questionnaire; BDI, Beck Depression Inventory; C-Qol, Cantril's ladder Quality of life Scale; DSEQ, Diabetes Self-Efficacy Outcomes Expectancies Questionnaire; GAD-7, General Anxiety Disorder questionnaire; SF-36 = (MOS)-SF-36, Medical Outcomes Study; SESM, Self-efficacy beliefs regarding self-management scale; SF-36, Short form health survey; SF-12, Short form health survey; STAI, State–Trait Anxiety Inventory; STAGI, State–Trait Anger Expression Inventory; SDSCA, Summary of Diabetes Self-Care Activities questionnaire; CES-D, The Centre for Epidemiological Studies-Depression; QoLd-II, Quality of Life Index—Diabetes III Version*.

### Limitations in reporting

Assumptions testing of statistical analysis methods were not reported in four studies (D'Eramo Melkus et al., 2010; Penckofer et al., 2012; Rosland et al., 2015; Stoop et al., 2015). Implications for policy were not addressed in six studies (Kuijer et al., 2007; Penckofer et al., 2012; Huang et al., 2015; Rosland et al., 2015; Stoop et al., 2015; Moncrieft et al., 2016) and implications for practice were not addressed in two studies (Kuijer et al., 2007; D'Eramo Melkus et al., 2010). Strengths and limitations were not addressed in one study (D'Eramo Melkus et al., 2010) and whether informed consent was obtained was not specified in two studies (Kuijer et al., 2007; Rosland et al., 2015). Obtainment of ethics approval was not specified in one study (Kuijer et al., 2007). The location of the intervention delivery was not addressed in two studies (Kuijer et al., 2007; Huang et al., 2015). In two studies the authors did not specify who collected the outcomes measures or whether the personal collecting the data were blind to group assignment (Huang et al., 2015; Rosland et al., 2015; Moncrieft et al., 2016). In one study, the care setting was not sufficiently described and previous articles needed to be accessed to obtain the missing information (Rosland et al., 2015).

Two studies were underpowered (D'Eramo Melkus et al., 2010; Moncrieft et al., 2016); another stated that it was underpowered, but did not provide information about the power calculations (Kuijer et al., 2007). In one trial information about power calculations were not provided in text (Stoop et al., 2015) but the previously published trial paper (Stoop et al., 2011) states that 80 individuals in both groups would be required to detect a moderate effect of 0.5 SD on the PHQ-9 and GAD-7, accounting for attrition, while only 46 were randomized in the RCT (Stoop et al., 2015).

### Meta-analysis

#### Depression outcomes

**Figure 2:**
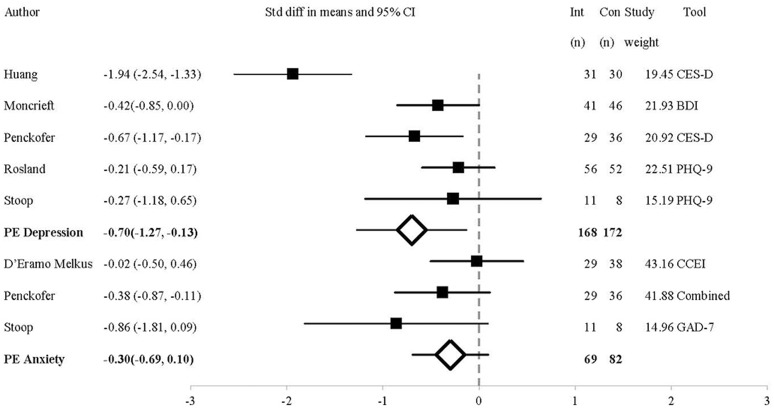
Forest Plot of Psychosocial Interventions on Depressive and Anxious Symptoms by Study. BDI, Beck Depression Inventory II; Combined, Study used a combination of tools to measure the outcome of interest; CCEI, Crown-Crisp Experiential Index; CES-D, Centre for Epidemiologic Studies-Depression; GAD-7, 7-item Generalized Anxiety Disorder questionnaire; PHQ-9, 9-item Patient Health Questionnaire; PE, Point Estimate.

Figure 2 shows the comparative efficacy of psychosocial interventions and UC on depressive symptoms. The post-intervention analysis included five studies. Outcome measures were the BDI (Moncrieft et al., 2016), CES-D (Penckofer et al., 2012; Huang et al., 2015) and PHQ-9 (Rosland et al., 2015; Stoop et al., 2015). The *SMD* = −0.70 *CI* = −1.27 to 0.13, indicating that psychosocial interventions have a medium-large effect of reducing depression over UC (*Z* = −2.4, *p* = 0.02, *Q* = 23.70 (4*df*), *I*^2^ = 83.18, *T*^2^ = 0.34, *T* = 0.58). One-study removed sensitivity analysis showed that removal of the study by Penckofer et al. (Kuijer et al., 2007) resulted in a non-significant difference between groups (*p* = 0.06).

At 3 month follow-up, the analysis included two studies using the CES-D (Penckofer et al., 2012; Huang et al., 2015). The *MD* = −8.18, *CI* = −10.90 to −5.46, *Z* = −5.89, *p* < 0.01, *Q* = 0.02 (1*df*), *I*^2^, *T*^2^, *T* = 0. Only one study assessed depressive symptoms at 6 months follow-up, and found that the psychosocial intervention did not influence depressive symptoms compared to UC (Rosland et al., 2015).

As the *Q* statistic indicated that the effect-size varied across studies post-intervention, we performed a subgroup analysis comparing outcomes between different depression measures (only the CES-D and PHQ-9 were compared as only one study used the BDI). In the two studies that used the CES-D (Penckofer et al., 2012; Huang et al., 2015), the *MD* = −7.25, *CI* = −10.01 to −4.49. Conversely, for the two studies that used the PHQ-9 (Rosland et al., 2015; Stoop et al., 2015), *MD* = −1.06, *CI* = −3.46 to −1.34. This shows that the effect size was higher in studies measuring depression using the CES-D compared to the PHQ-9. This may be why significant differences were also seen at 3 month follow-up, in the studies that used the CES-D (Penckofer et al., 2012; Huang et al., 2015), but not in the study with the 6 month follow-up, in which the PHQ-9 was used (Rosland et al., 2015). The four studies evenly contributed to the reported outcomes (20–27% each).

#### Anxiety outcomes

As shown in Figure 2, the analysis of anxiety symptoms post-intervention includes three studies using the STAI (composite score of STAI-T and STAI-S) (Penckofer et al., 2012), CCEI (D'Eramo Melkus et al., 2010), and GAD-7 (Stoop et al., 2015). At post-intervention, the *SMD* = −0.30, *CI* = −0.69 to 0.10 (*Z* = −1.73, *p* = 0.08, *Q* = 2.69(2*df*), *I*^2^ = 25.67, *T*^2^ = 0.03, *T* = 0.18). One trial contributed 14%, (Stoop et al., 2015) while two contributed over 38 and 36% (D'Eramo Melkus et al., 2010; Penckofer et al., 2012). Therefore, two studies are largely responsible for the findings. One-study removed sensitivity analysis showed that removal of the study by D'Eramo Melkus et al. (Michie et al., 2011) resulted in a significant difference between groups (*p* = 0.03).

At 3 month follow-up two studies measured anxiety outcomes, one using the CCEI (D'Eramo Melkus et al., 2010) and the other using the STAI (Penckofer et al., 2012), the *SMD* = −0.61, *CI* = −0.92 to −0.31, *Z* = −3.93, *p* = 0.00, *Q* = 0.15 (1*df*), *I*^2^*, T*^2^, and *T* = 0. Thus, psychosocial interventions decreased anxious symptoms at 3 months follow-up compared to UC.

At 6 month follow-up two studies measured anxiety outcomes, one using the CCEI (D'Eramo Melkus et al., 2010) and the other using the GAD-7 (Stoop et al., 2015), *SMD* = −0.47, *CI* = −0.98 to 0.03, *Z* = −1.85, *p* = 0.06, *Q* = 1.04 (1*df*), *I*^2^ = 3.81, *T*^2^ = 0.01, *T* = 0.08, demonstrating that the decrease in anxious symptoms was not sustained at 6 month follow-up.

#### Quality of life outcomes

**Figure 3:**
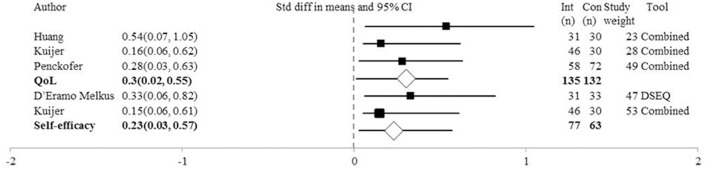
Forest Plot of Psychosocial Interventions on Quality of Life and Self-Efficacy Symptoms by Study. DSEQ, Diabetes Self-Efficacy Outcomes Expectancies Questionnaire; Combined, Study used a combination of tools to measure the outcome of interest; PE, Point Estimate; QoL, Quality of life.

As shown in Figure 3, three studies measured QoL at post-intervention using the SF-36 (Huang et al., 2015), SF-12 (Kuijer et al., 2007; Penckofer et al., 2012), C-QoL (Kuijer et al., 2007) and QoLd-III (Penckofer et al., 2012). A composite mean score was used. The *SMD* = 0.30, *CI* = 0.06–0.55, *Z* = 2.44, *p* = 0.02, *Q* = 1.20 (3*df*), *I*^2^, *T*^2^*, T* = 0. One-study removed sensitivity analysis showed that removal of Huang et al. (Penckofer et al., 2012), (*SMD* = 0.24, *p* = 0.10) or Penckofer et al. (Kuijer et al., 2007), (*SMD* = 0.39, *p* = 0.08) resulted in a non-significant difference between groups. One study contributed 49% (Penckofer et al., 2012) and two studies contributed 28% (Kuijer et al., 2007) and 23% (Huang et al., 2015) of the weight of the results.

At 3 month follow-up, the analysis included three studies using the SF-36 (Huang et al., 2015), MOS-SF-36 (D'Eramo Melkus et al., 2010) SF-12 and QoLd-III (Penckofer et al., 2012). The *SMD* = 0.52, *CI* = 0.10–0.95, *Z* = 2.40, *p* = 0.02, *Q* = 4.95 (2*df*), *I*^2^ = 59.62, *T*^2^ = 0.08, *T* = 0.29. Only one study assessed QoL outcomes at 6 month follow-up (Kuijer et al., 2007).

#### Self-efficacy outcomes

Figure 3 shows that at post-intervention the analysis of self-efficacy (relating to disease management) outcome was completed in two studies, one using the DSEQ (D'Eramo Melkus et al., 2010) and the other using a SESM and the SDSCA (Kuijer et al., 2007). The *SMD* = 0.23, *CI* = −0.11 to 0.57, *Z* = 1.35, *p* = 0.18, *Q* = 0.27 (1*df*), *I*^2^, *T*^2^, and *T* = 0. Therefore, the psychosocial interventions do not appear to influence beliefs of self-efficacy. Both studies contributed equally to the outcomes; 47% (Kuijer et al., 2007) vs. 53% (D'Eramo Melkus et al., 2010). Only one study (D'Eramo Melkus et al., 2010) explored self-efficacy at 3 month follow-up and found no significant difference between groups. At 6 month follow-up, two studies assessed self-efficacy (Kuijer et al., 2007; D'Eramo Melkus et al., 2010) and did not find a significant difference between groups, *SMD* = 0.19, *CI* = −0.19 to 0.57, *Z* = 1.0, *p* = 0.32, *Q* = 0.57 (1*df*), *I*^2^, *T*^2^, *T* = 0.

### Other outcomes

BMI was measured in two studies post-intervention (D'Eramo Melkus et al., 2010; Huang et al., 2015), *MD* = −1.46, *CI* = −4.64 to 1.72, *Z* = −0.90, *p* = 0.37, *Q* = 0.02 (1*df*), *I*^2^*, T*^2^, and *T* = 0. HbA1c was measured in five studies post-intervention (D'Eramo Melkus et al., 2010; Penckofer et al., 2012; Huang et al., 2015; Rosland et al., 2015; Moncrieft et al., 2016), *MD* = −0.15, *CI* = −0.65 to 0.06, *Z* = −0.58, *p* = 0.56, *Q* = 4.27 (4df), *I*^2^ = 6.32, *T*^2^ = 0.02 and *T* = 0.15. Social support was measured in two studies using the DCP post-intervention (D'Eramo Melkus et al., 2010; Rosland et al., 2015), *MD* = 0.09, *CI* = −0.36 to −0.53, *Z* = 0.38, *p* = 0.71, *Q* = 0.44 (1 *df*), *I*^2^*, T*^2^, and *T* = 0. Therefore, psychosocial interventions did not appear to influence BMI, HbA1c or social support post-intervention. No changes were seen at follow-up (not reported) for BMI or HbA1c and only one study assessed social support at follow-up. FBG was measured in three studies (D'Eramo Melkus et al., 2010; Penckofer et al., 2012; Huang et al., 2015) and at post-intervention the *MD* = −26.18, *CI* = −51.58 to −0.79, *Z* = −2.02, *p* = 0.04, *Q* = 1.17 (2*df*), *I*^2^*, T*^2^, and *T* = 0. Therefore, psychosocial interventions appeared to decrease fasting glucose. Similar effects were seen at 3 month follow-up, *MD* = −29.69, *CI* = −56.24 to −3.15, *Z* = −2.19, *p* = 0.03, *Q* = 1.49 (2*df*), *I*^2^*, T*^2^, and *T* = 0. Only one study (D'Eramo Melkus et al., 2010) explored fasting glucose at 6 month follow-up, and found no significant difference between groups.

## Discussion

This systematic review and meta-analysis found that psychosocial interventions, compared to UC, reduce depressive symptoms in individuals with diabetes and that these effects persist at 3 month follow-up. The magnitude of the standardized mean difference reflects a medium-large benefit of the intervention. Subgroup analysis of depression outcomes show that the effect-size was larger in studies measuring depression using the CES-D compared to the PHQ-9. The efficacy and accuracy of the CES-D and PHQ-9, in comparison with clinical interview, has previously been assessed a study involving 185 individuals with type II diabetes (Khamseh et al., 2011). The authors reported that while using the PHQ-9, 47.6% of individuals were diagnosed with major depressive disorder, while 61.62% were diagnosed while using the CES-D. These results indicate that the CES-D may be more sensitive than the PHQ-9 at identifying depressive symptoms in people with diabetes.

Anxiety symptoms were not found to decrease post-intervention, but were decreased at 3 month follow-up. This is likely to reflect that fact that the 3 month assessment did not include the study using the CCEI measure of somatic anxiety (D'Eramo Melkus et al., 2010), which is arguably quite different from state, trait and generalized anxiety. Indeed, one-study removed analysis showed that removal of the aforementioned study using the CCEI measure (D'Eramo Melkus et al., 2010) resulted in a small-medium benefit of the intervention between groups directly post-intervention. Additionally, there was significant variation between the psychosocial interventions, including the mode of delivery, the individual delivering the intervention, the setting, frequency and duration of session and most likely the therapeutic alliance. These differences likely contribute to the statistical heterogeneity seen in effect sizes for some outcomes. In addition, the results of our study indicate that psychosocial interventions may offer a small benefit in terms of QoL.

The beneficial effect seen in depressive symptoms is consistent with a previous meta-analysis of psychosocial interventions with individuals with coronary heart disease that found a reduction in depressive and anxious symptoms (Ski et al., 2015). However, in this previous study, psychosocial interventions were seen to have a small benefit for depressive symptoms (*SMD* = 0.15) and a medium to large benefit for anxiety symptoms (*SMD* = 0.76). Our current results indicate that the effect of psychosocial intervention on reducing depression and anxiety are greater in a DM population compared to a coronary heart disease population. As we reported *SMD* rather than mean difference in the current meta-analysis, the clinical significance of these effect sizes is not inherently meaningful. The effect size can be interpreted as the probability of patients in the intervention group improving over and above a patient receiving usual care (Faraone, 2008). Our effect size for depressive symptoms was small and for anxiety symptoms it was medium to large, meaning that the decrease in depressive symptoms resulting from psychosocial interventions is likely to be small, and the decrease in anxiety symptoms resulting from psychosocial interventions is likely to be medium to large. There are a number of limitations to our meta-analysis. A number of the primary studies are characterized by a small sample size. There was also heterogeneity in terms of the data collection schedules in the primary studies, and some trials did not collect some outcomes directly post-intervention; only at baseline and follow-up. There was also significant heterogeneity between the studies in terms of what constituted a psychosocial intervention, and often the interventions were not sufficiently described to facilitate replication. This likely reflects the developing nature of this field. Indeed, very few studies were identified that investigated the effects of psychosocial interventions, compared to UC, in a DM population. We see the small number of identified studies in the current meta-analysis as a testimony to the need for further research in this field. Indeed, previously published meta-analyses have similarly included a limited number of studies due to the lack of research in the field on inquiry (Charidimou et al., 2013; Cleland et al., 2013; Menon et al., 2013; Goyal et al., 2016). Depression symptoms were only assessed in five studies, anxiety and QoL in three, and self-efficacy in two studies. Thus the reported findings should be interpreted with caution. A final limitation of the current meta-analysis is that in five studies (Kuijer et al., 2007; D'Eramo Melkus et al., 2010; Huang et al., 2015; Rosland et al., 2015; Moncrieft et al., 2016), it was not specified whether some participants received antidepressant or anxiolytic treatment during the course of the intervention. In one study (Penckofer et al., 2012), participants who reported using psychoactive medications prior to entry into the study remained on these while participating in the study, and the authors highlighted that there was no significant difference in self-reported use of psychoactive medications between the intervention and control groups. In another study (Stoop et al., 2015) the use of pharmacotherapy was not an exclusion criterion and the authors noted that they did not have information about any potential dose changes in participants using pharmacotherapy and therefore were unable to control for this in the analyses. Thus, the potential confounding role of pharmaceutical anti-depressant medication in the primary studies is largely unknown.

While some studies have assessed the impact of lifestyle interventions and psychological interventions on wellbeing in people with DM, to our knowledge this is the first meta-analysis to assess the effectiveness of psychosocial interventions, being interventions with both a psychological and social component, in a DM population. The results of our meta-analysis indicate that various psychosocial interventions appear effective for the management of depressive symptoms and may improve QoL. In future studies it would be useful to include a cost-benefit analysis to determine if such psychosocial interventions are cost-effective, compared to usual care. The limited number of studies in this meta-analysis highlights the need for additional research in this field, to confirm or refute the current encouraging findings and to explore what type of psychosocial interventions may be most effective.

## Author contributions

MP conceived the study including data sources and search strategy, conducted the systematic search, performed study selection, extracted data, performed data synthesis and wrote the manuscript. DT conceived the study including data sources and search strategy and critically appraised the manuscript. DC conceived the study. ZJ performed study selection and extracted data. CS conceived the study including data sources and search strategy and critically appraised the manuscript. All authors take responsibility for the contents of this article.

### Conflict of interest statement

The authors declare that the research was conducted in the absence of any commercial or financial relationships that could be construed as a potential conflict of interest.
