# Supplementary material for: Psychosocial Interventions and Wellbeing in Individuals with Diabetes Mellitus: A Systematic Review and Meta-Analysis
Source: Front Psychol. 2017 Dec 5;8:2063. doi: 10.3389/fpsyg.2017.02063 (PMC5723413; doi:10.3389/fpsyg.2017.02063)

Supplementary Figure 1: Funnel plot showing publication bias for studies assessing depressive symptoms

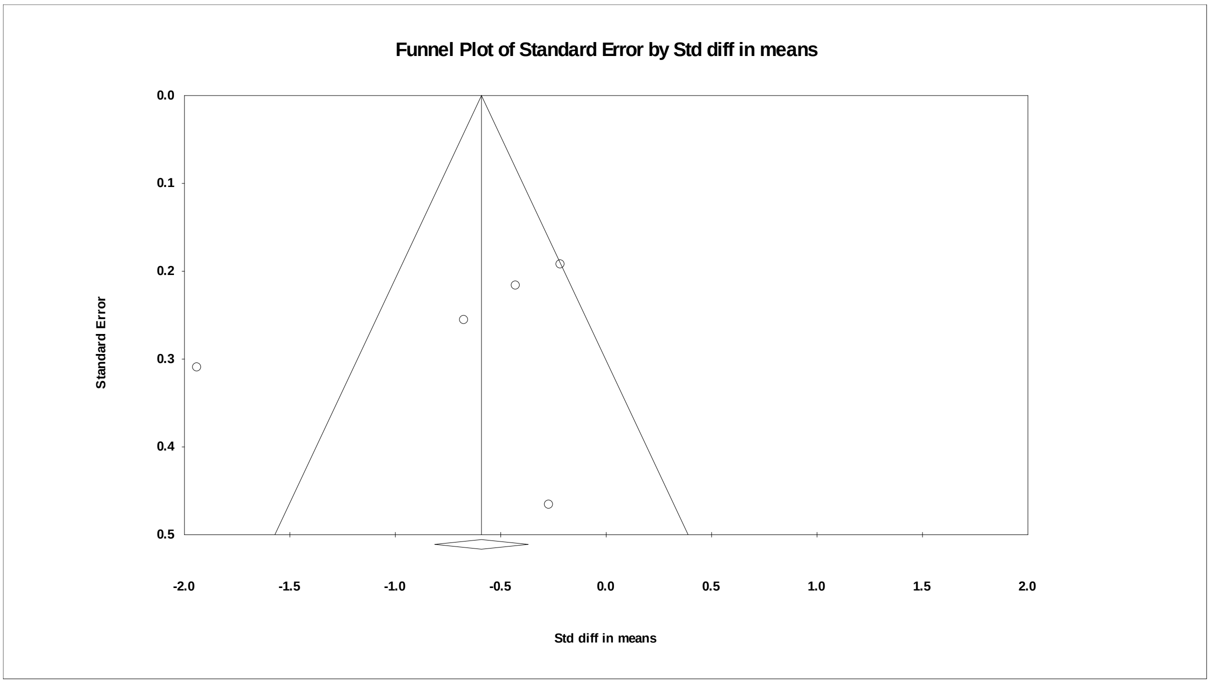

Supplement: Supplementary file 1 [file Image1.pdf]
